# Strategic Orthograde Biodentine Obturation and Regenerative Surgical Management of a Persistent Radicular Cyst Refractory to Nonsurgical Endodontic Therapy: A One-Year Follow-Up Case Report

**DOI:** 10.7759/cureus.117016

**Published:** 2026-09-27

**Authors:** Suraj Khose, Rushikesh Mahaparale, Prasad Shirke, Pranali Suryavanshi, Trupti Kadam, Abhijeet Taksale, Siddhartha Varma

**Affiliations:** 1 Department of Conservative Dentistry and Endodontics, School of Dental Sciences, Krishna Vishwa Vidyapeeth (Deemed to be University), Karad, IND; 2 Department of Periodontology, School of Dental Sciences, Krishna Vishwa Vidyapeeth (Deemed to be University), Karad, IND

**Keywords:** apicoectomy, biodentine, cone-beam computed tomography, platelet-rich fibrin, radicular cyst, tooth injuries

## Abstract

Large cyst-like periapical lesions may persist despite adequate nonsurgical endodontic therapy and often present a significant therapeutic challenge. The present case report highlights the effective management of a persistent radicular cyst associated with traumatized maxillary anterior teeth using orthograde Biodentine® obturation and regenerative endodontic surgery. A 24-year-old male patient with a previous history of road traffic injury presented with Ellis class IV fractures involving teeth 11, 12, 21, and 22 and associated extensive periapical radiolucent pathology. Nonsurgical root canal treatment was performed; however, persistent exudation from teeth 21 and 22 persisted despite repeated applications of calcium hydroxide and triple antibiotic paste. While teeth 11 and 12 were conventionally obturated using gutta-percha and sealer, teeth 21 and 22 underwent orthograde Biodentine^®^ obturation prior to surgical intervention. Surgical management included lesion curettage, apicoectomy, and regenerative reconstruction of the osseous defect using bone graft, injectable platelet-rich fibrin, leukocyte platelet-rich fibrin, and a barrier membrane. Following satisfactory healing, definitive prosthetic rehabilitation was completed using a digital workflow and full-coverage restorations. One-year follow-up demonstrated resolution of clinical symptoms, substantial radiographic bone regeneration, and satisfactory esthetic and functional outcomes. Orthograde Biodentine^®^ obturation combined with regenerative surgical management may be a useful treatment option for persistent periapical lesions that fail to respond to prolonged nonsurgical therapy.

## Introduction

Periapical lesions are inflammatory disorders of the periradicular tissues that develop secondary to pulpal necrosis and intracanal microbial infection. These lesions represent the host immune response to persistent intracanal microorganisms and their by-products and may manifest as periapical granulomas, abscesses, or cysts [1]. Although conventional nonsurgical endodontic treatment is regarded as the preferred treatment approach for teeth with apical periodontitis, the management of large cyst-like periapical lesions continues to pose a significant clinical challenge, particularly when associated with long-standing trauma and extensive periapical bone destruction [1,2].

Successful nonsurgical endodontic therapy depends on thorough disinfection of the root canal system and prevention of subsequent reinfection. Intracanal medicaments such as calcium hydroxide are widely used because of their antimicrobial activity and potential to support periapical healing [3]. In persistent infections, triple antibiotic paste (TAP) has been advocated as an adjunctive medicament owing to its broad-spectrum antibacterial properties [4]. However, certain lesions remain refractory to conventional treatment despite meticulous chemomechanical preparation and repeated intracanal medication, resulting in persistent exudation, delayed healing, and failure to achieve definitive obturation [3,4].

In such situations, endodontic surgery serves as an important tooth-preserving treatment modality. Surgical intervention enables direct access to the pathological lesion, facilitates removal of infected periapical tissues, eliminates apical ramifications and potential extraradicular irritants, and promotes favorable periapical healing. Recent advances in surgical techniques, biomaterials, and regenerative strategies have substantially improved the predictability and long-term success of endodontic surgical procedures [5].

Biodentine® (Septodont, Saint-Maur-des-Fossés, France) is a calcium silicate-based bioactive material that has gained considerable attention in contemporary endodontics because of its excellent sealing ability, biocompatibility, bioactivity, and capacity to stimulate hard tissue formation [6]. Owing to its favorable physicochemical and biological properties, Biodentine® has been successfully employed in an array of clinical applications, including perforation repair, apexification, resorption management, pulp therapy, and root-end filling procedures. Nevertheless, the pre-surgical orthograde use of Biodentine® as a definitive obturation material prior to surgical endodontic intervention has been infrequently reported, despite its potential advantages in minimizing surgical complexity and reducing the risk of blood-field contamination during root-end filling procedures [6,7].

The present case report describes pre-surgical use of orthograde Biodentine® obturation in conjunction with regenerative surgical management for a persistent radicular cyst involving traumatized maxillary anterior teeth that remained refractory to prolonged nonsurgical endodontic therapy, including calcium hydroxide and TAP medication. Subsequent prosthetic rehabilitation and favorable clinical and radiographic outcomes following one year of follow-up are also presented.

## Case presentation

A 24-year-old male patient presented to the clinics of conservative dentistry and endodontics with a chief complaint of fractured upper front teeth and a desire for esthetic rehabilitation. The patient recalled a history of road traffic accident (RTA)-related trauma to the maxillary anterior region approximately three years prior to presentation. His medical history was non-contributory, and no extraoral abnormalities were detected on clinical examination.

Intraoral examination revealed fractures involving teeth 11, 12, 21, and 22 (Figure 1).

**Figure 1:**
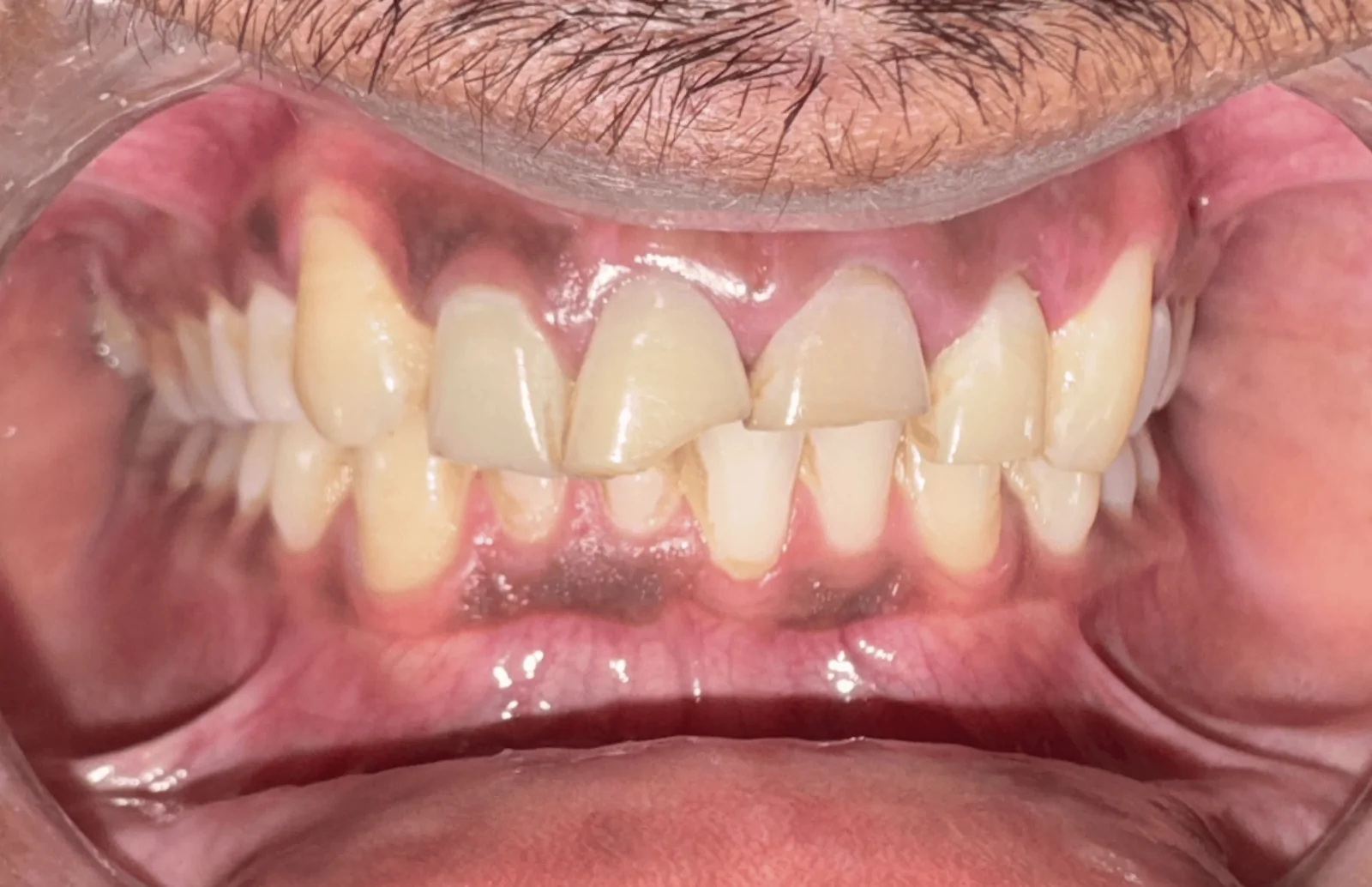
Preoperative intraoral clinical photograph showing Ellis class IV fractures involving teeth 11, 12, 21, and 22.

The affected teeth were nonresponsive to electric and cold pulp testing, and no tenderness to percussion was elicited. Based on the clinical examination, the fractures were classified as Ellis class IV involving teeth 11, 12, 21, and 22. Radiographic examination using intraoral periapical radiographs demonstrated periapical radiolucencies associated with the maxillary anterior teeth (Figure 2).

**Figure 2:**
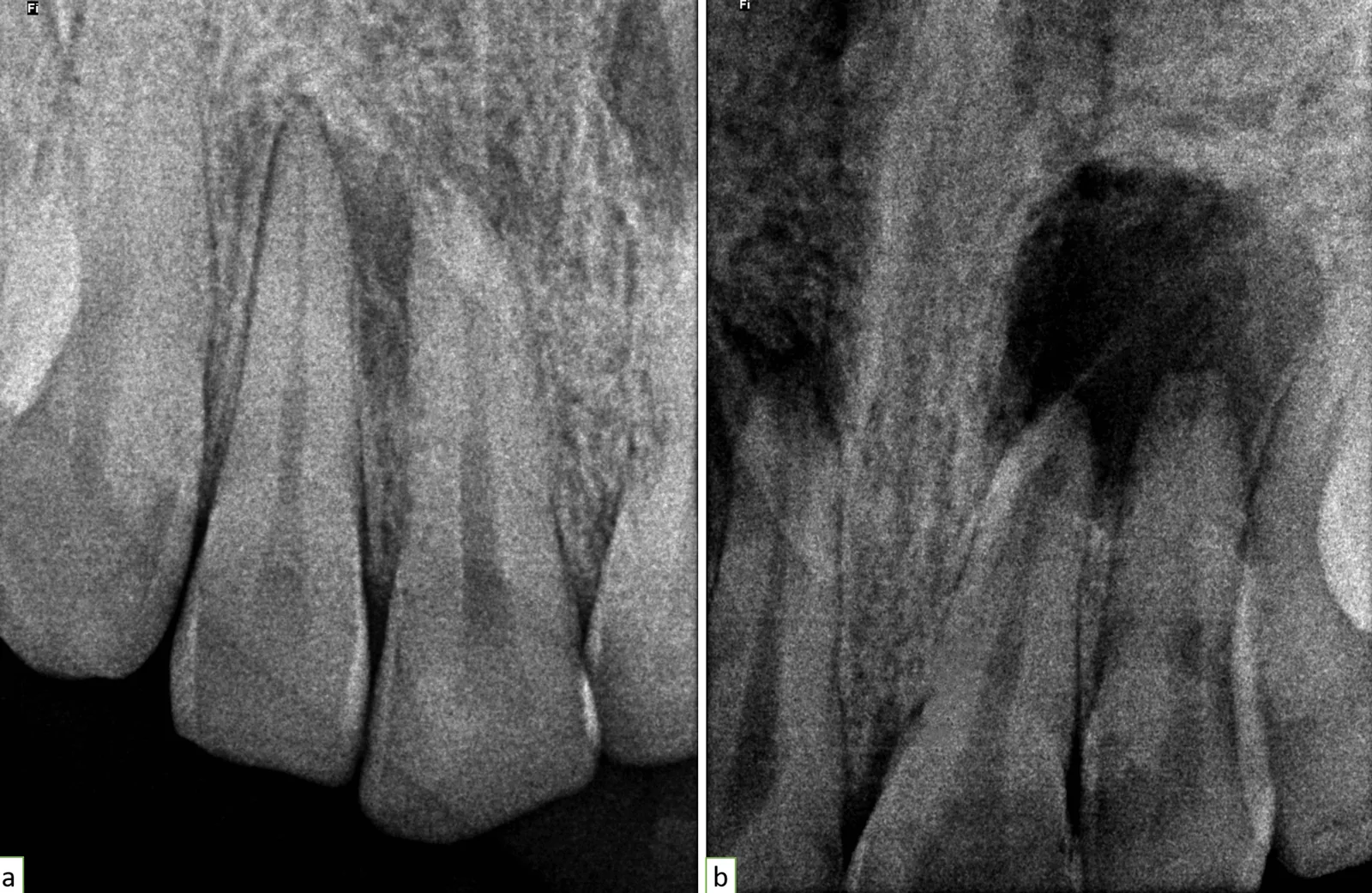
Preoperative intraoral periapical radiographs of the maxillary anterior region demonstrating associated periapical radiolucencies. (a) 11-12 region. (b) 21-22 region.

Cone-beam computed tomography (CBCT) revealed a well-defined periapical radiolucency involving the apices of teeth 11, 12, 21, and 22. The lesion measured approximately 12.9 mm buccopalatally, 12.6 mm superoinferiorly, and 11.9 mm mesiodistally, with associated periapical bone destruction (Figure 3).

**Figure 3:**
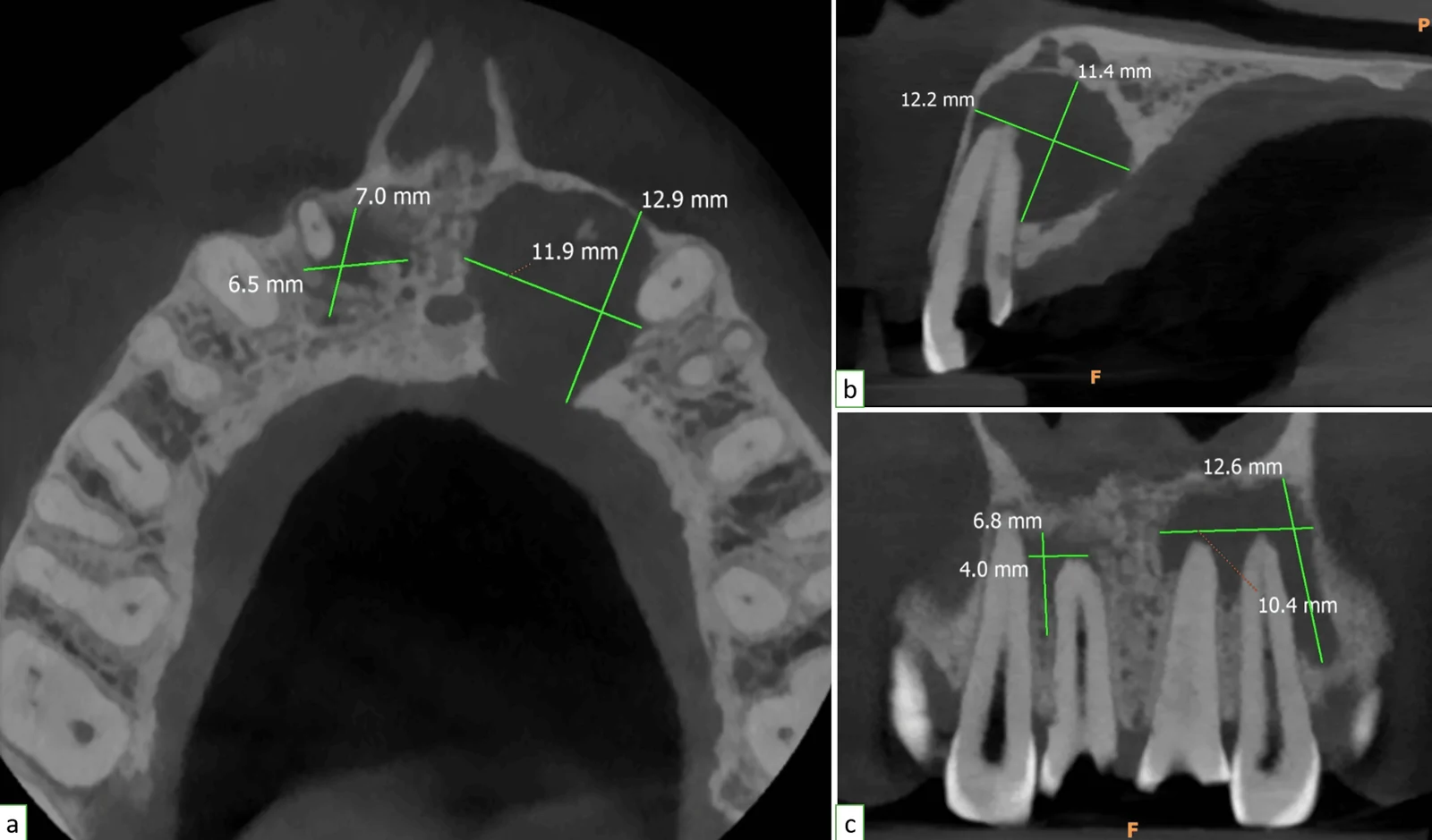
Preoperative cone-beam computed tomography images demonstrating a large cyst-like periapical radiolucent lesion involving the maxillary anterior region. (a) Axial view showing the mesiodistal and buccopalatal extent of the lesion. (b) Sagittal view demonstrating periapical bone destruction associated with the affected teeth. (c) Coronal view illustrating the superior-inferior extent of the lesion and involvement of the maxillary anterior periapical region.

The clinical and radiographic findings were consistent with pulp necrosis and asymptomatic apical periodontitis associated with a large cyst-like periapical lesion involving teeth 21 and 22.

Nonsurgical root canal treatment was initiated for teeth 11, 12, 21, and 22 under local anesthesia. Endodontic procedures were performed under rubber dam isolation using premolar-to-premolar clamps to maintain an isolated operative field and minimize salivary contamination. Access cavities were prepared, and working lengths were established using an electronic apex locator and confirmed radiographically (Figure 4).

**Figure 4:**
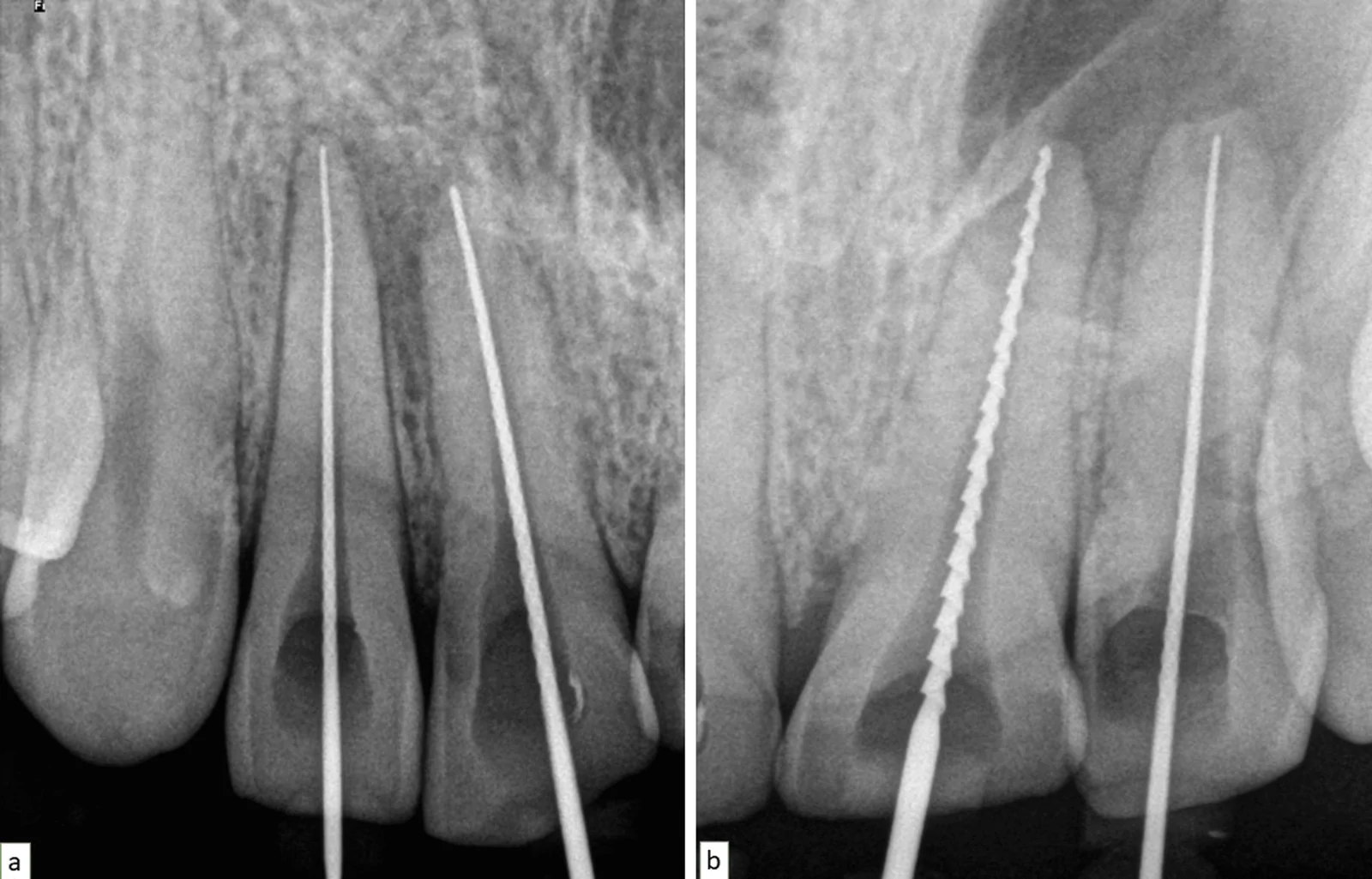
Radiographic confirmation of working lengths established using an electronic apex locator during endodontic treatment. (a) 11 and 12. (b) 21 and 22.

Cleaning and shaping were performed using the ProTaper Gold rotary system (Dentsply Sirona, Ballaigues, Switzerland) up to size F2. Calcium hydroxide intracanal medicament was placed and renewed at 14-day intervals on two occasions. However, persistent exudation and lack of clinical improvement were observed. Subsequently, TAP was placed as an intracanal medicament and renewed at 21-day intervals on two occasions. Despite repeated calcium hydroxide and TAP dressings, persistent exudation from teeth 21 and 22 precluded definitive obturation and suggested failure of nonsurgical therapy. Conversely, teeth 11 and 12 demonstrated favorable healing and were obturated conventionally using gutta-percha and MTA Fillapex sealer (Angelus, Londrina, Brazil) (Figure 5).

**Figure 5:**
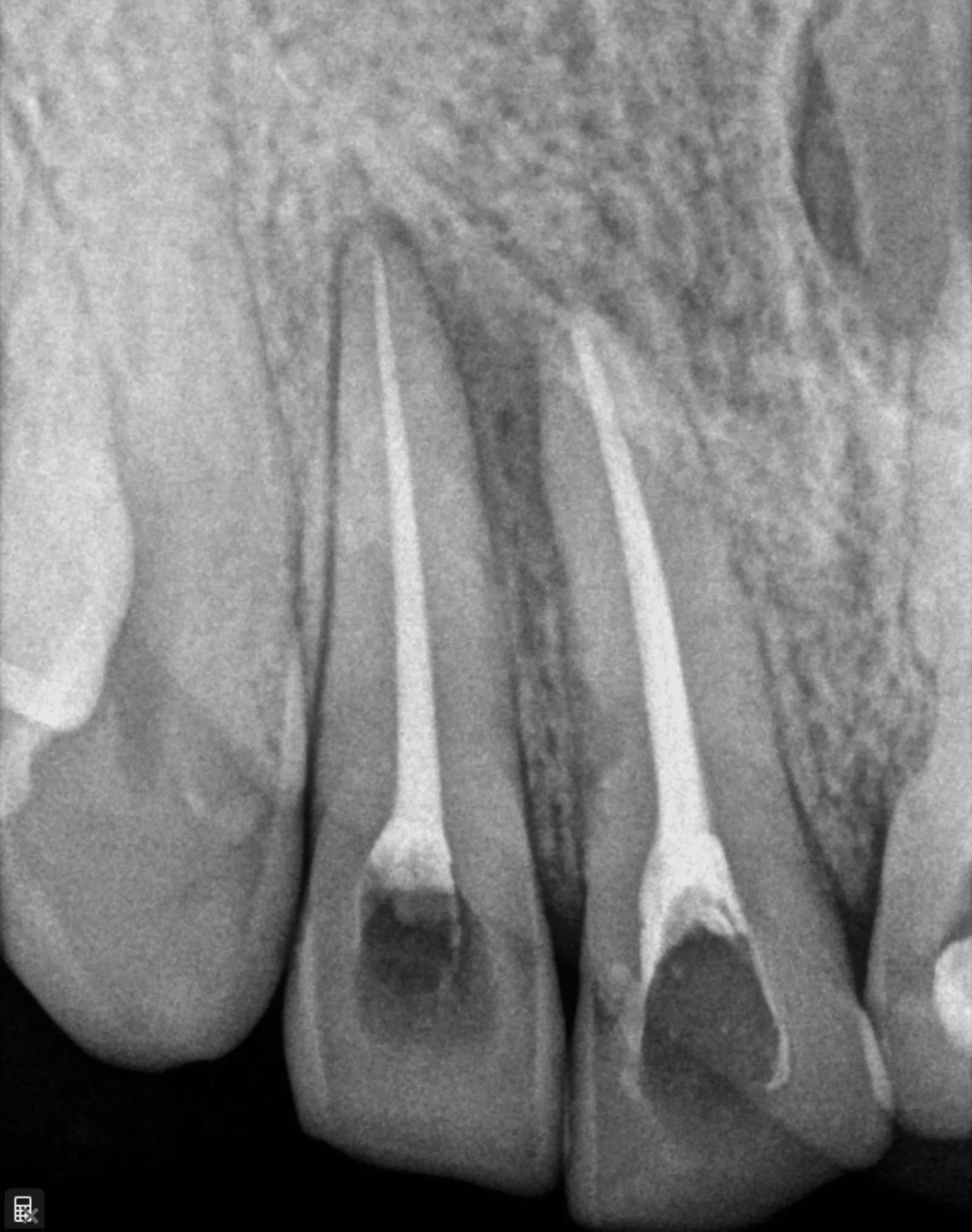
Post-obturation intraoral periapical radiograph demonstrating conventional root canal obturation of teeth 11 and 12 using gutta-percha and MTA Fillapex sealer.

Given the persistent infection associated with teeth 21 and 22, surgical management was deemed necessary. In view of the planned surgical intervention, teeth 21 and 22 underwent orthograde Biodentine® obturation one day prior to surgery to allow adequate setting of the material prior to surgical intervention (Figure 6).

**Figure 6:**
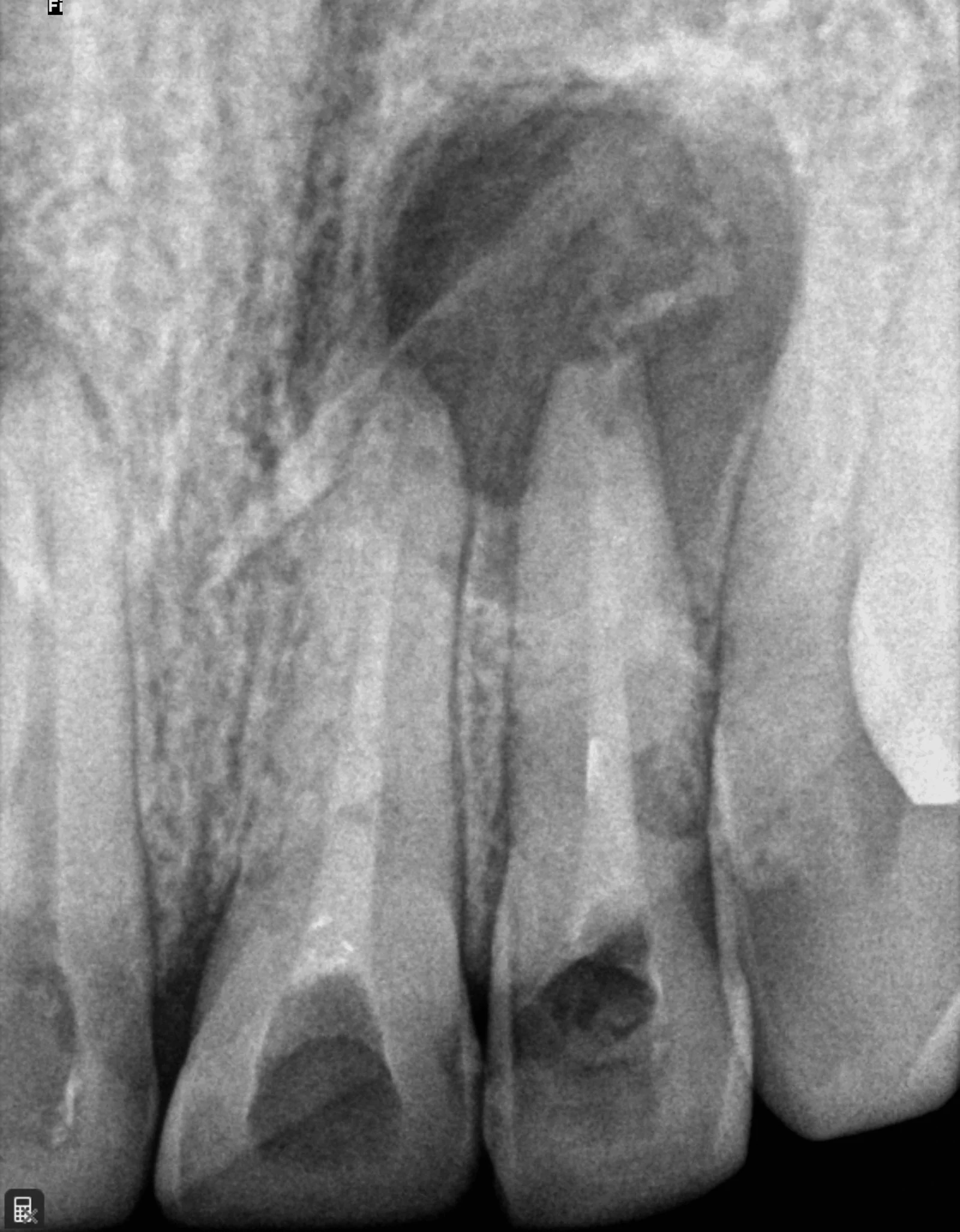
Intraoral periapical radiograph demonstrating orthograde Biodentine® obturation of teeth 21 and 22 performed one day prior to surgical intervention.

This approach was intended to reduce surgical complexity, shorten operative time, and eliminate the need for retrograde root-end filling in a potentially contaminated surgical field.

Biodentine® was prepared by adding five drops of the supplied liquid to the powder-filled capsule, followed by mechanical trituration at 4000-4200 rpm for 30 seconds in an amalgamator, according to the manufacturer’s instructions. The prepared material was subsequently delivered into the root canals using an amalgam carrier and condensed incrementally with a GDC root canal plugger (GDC Dental, Punjab, India) to ensure adequate adaptation and obturation of the canal space.

Under local anesthesia, a full-thickness trapezoidal mucoperiosteal flap was reflected in the maxillary anterior region. Cortical bone overlying the lesion was removed using a slow-speed round surgical bur #8 under copious saline irrigation to expose the periapical defect (Figure 7).

**Figure 7:**
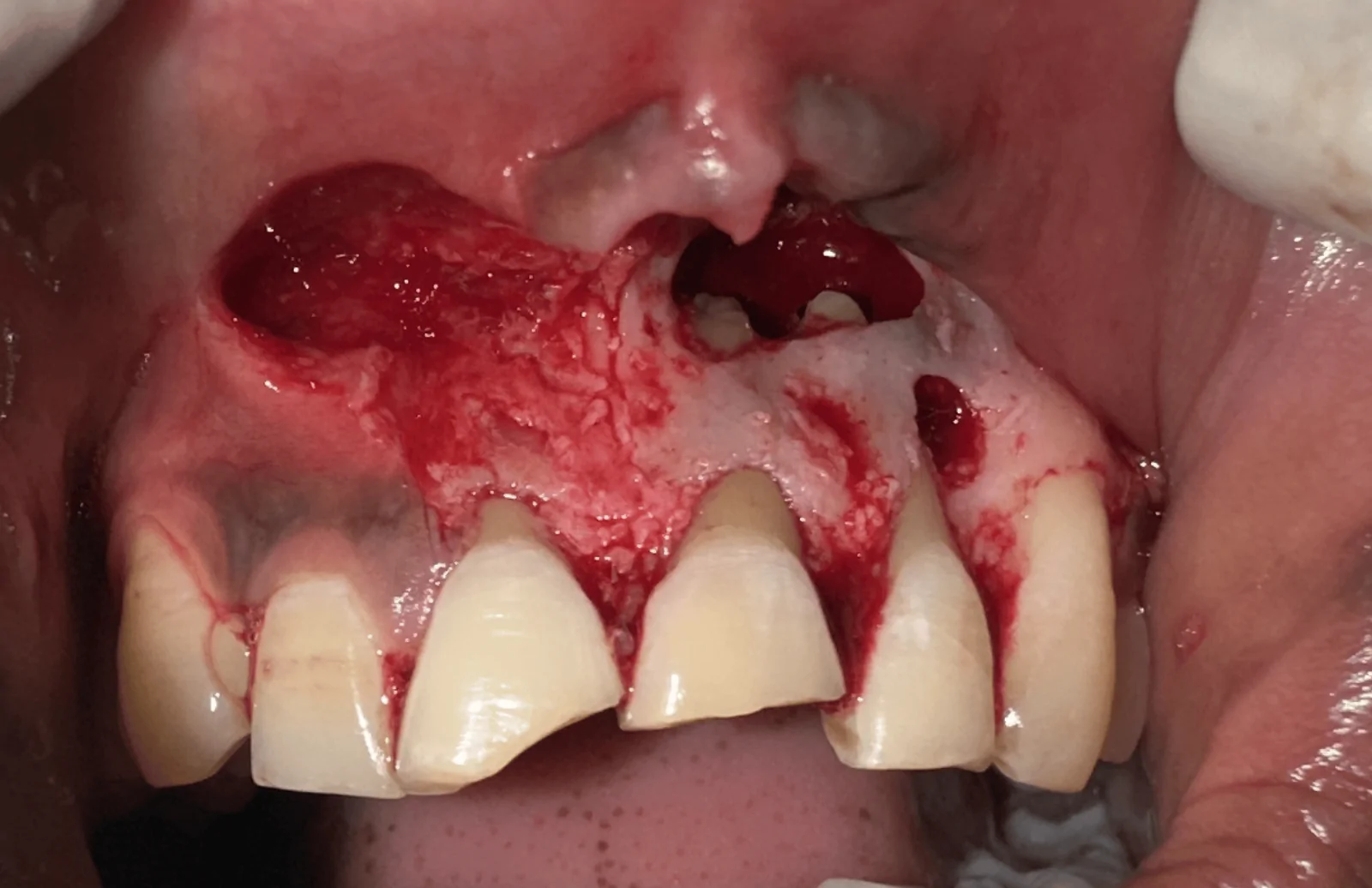
Intraoperative clinical photograph showing reflection of a full-thickness trapezoidal mucoperiosteal flap with visualization of the periapical osseous defect associated with the lesion.

Thorough curettage of the pathological tissue was performed, followed by apicoectomy with resection of approximately 3 mm of the root apex at a 0° bevel angle (Figure 8).

**Figure 8:**
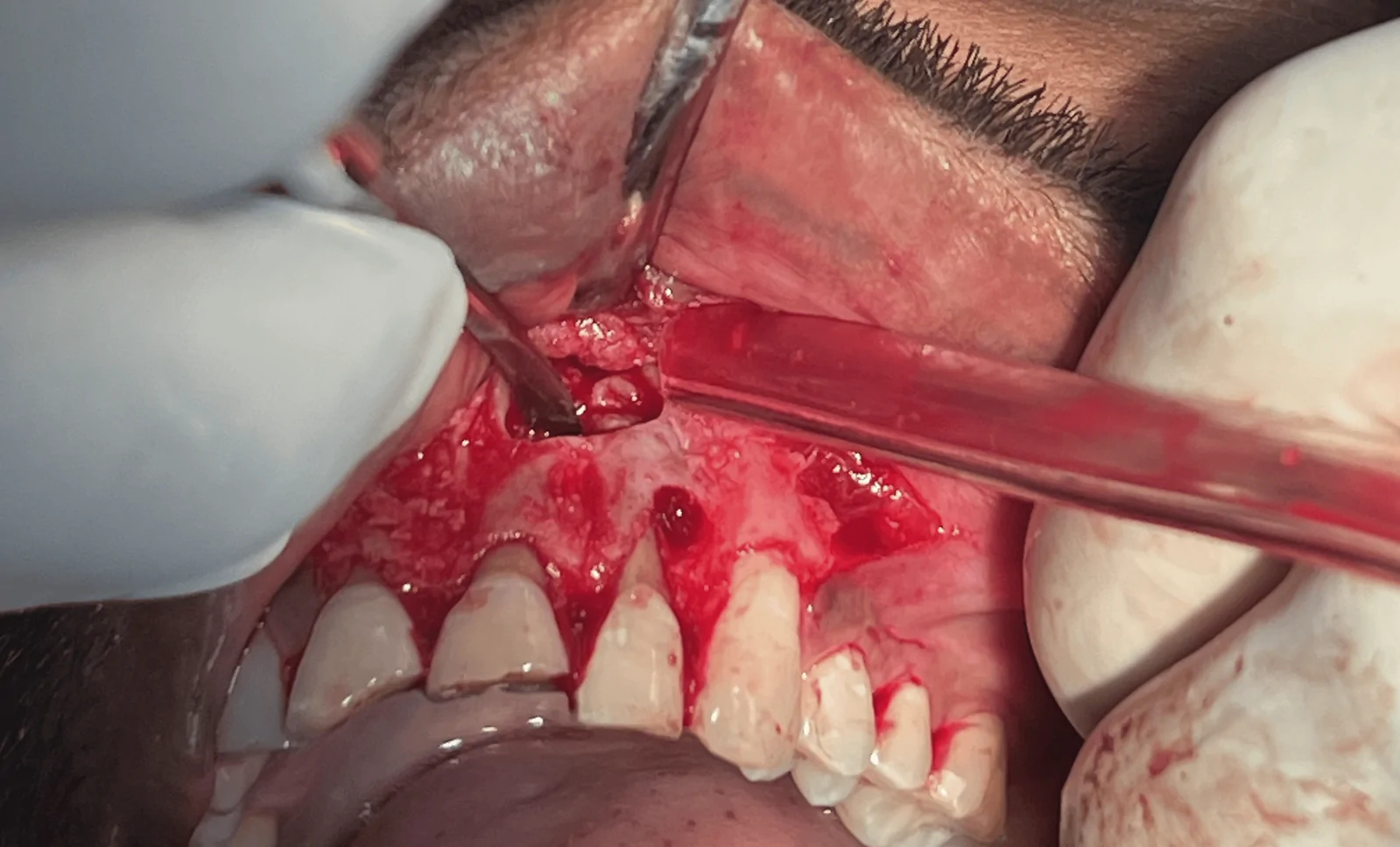
Intraoperative clinical photograph demonstrating complete curettage of the pathological tissue.

The excised periapical tissue was sent for histopathological examination. Histological evaluation revealed a cystic cavity lined by non-keratinized stratified squamous epithelium with an underlying chronically inflamed fibrous connective tissue wall, confirming the diagnosis of a radicular cyst (Figure 9).

**Figure 9:**
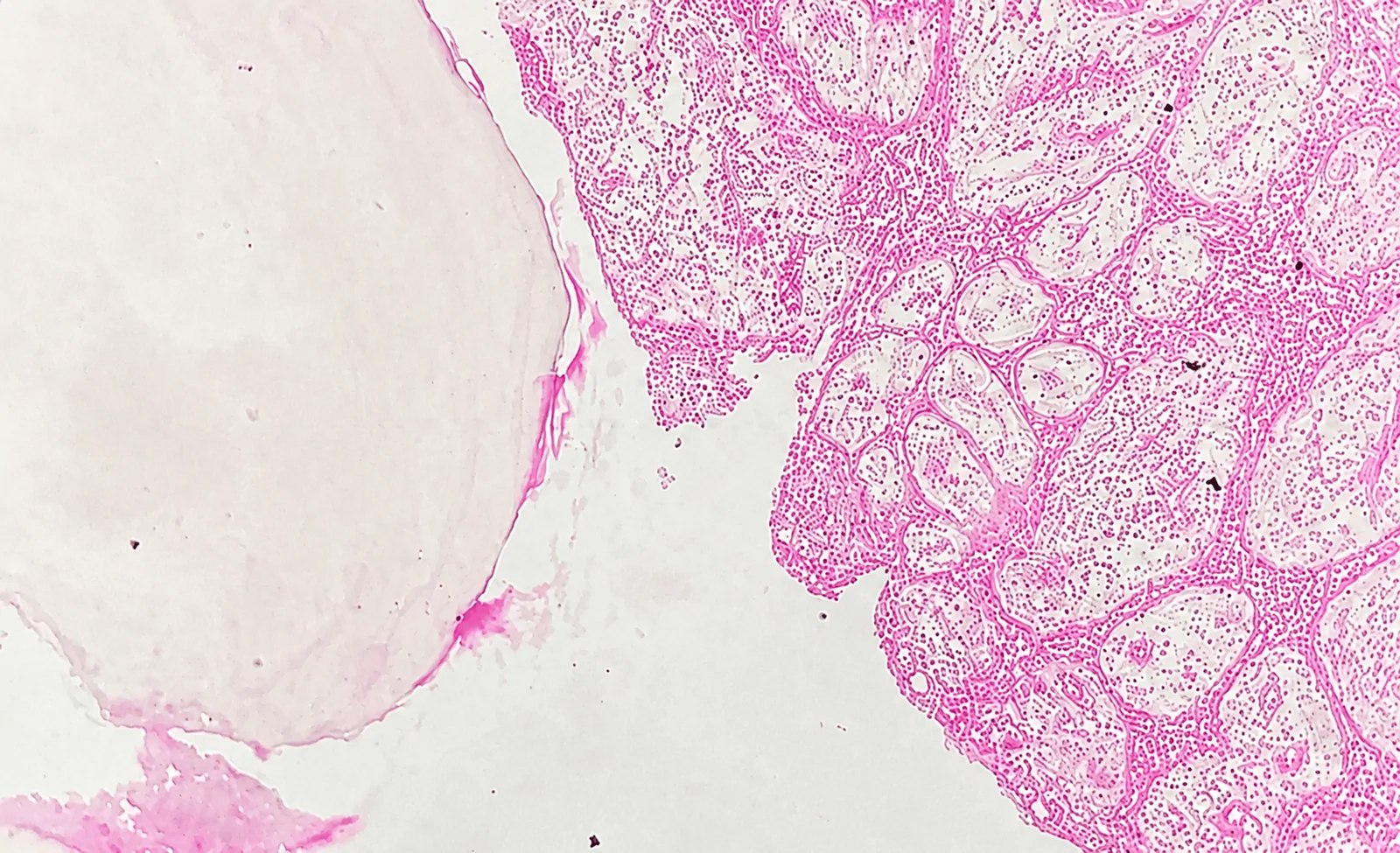
Histopathological photomicrograph showing features consistent with a radicular cyst (H&E stain, ×40).

Regenerative management of the osseous defect was subsequently performed. Injectable platelet-rich fibrin (I-PRF) was prepared at 700 rpm for three minutes and combined with Osseograft® (Advanced Biotech Products Pvt. Ltd., Chennai, India) bone graft material to obtain sticky bone (Figure 10).

**Figure 10:**
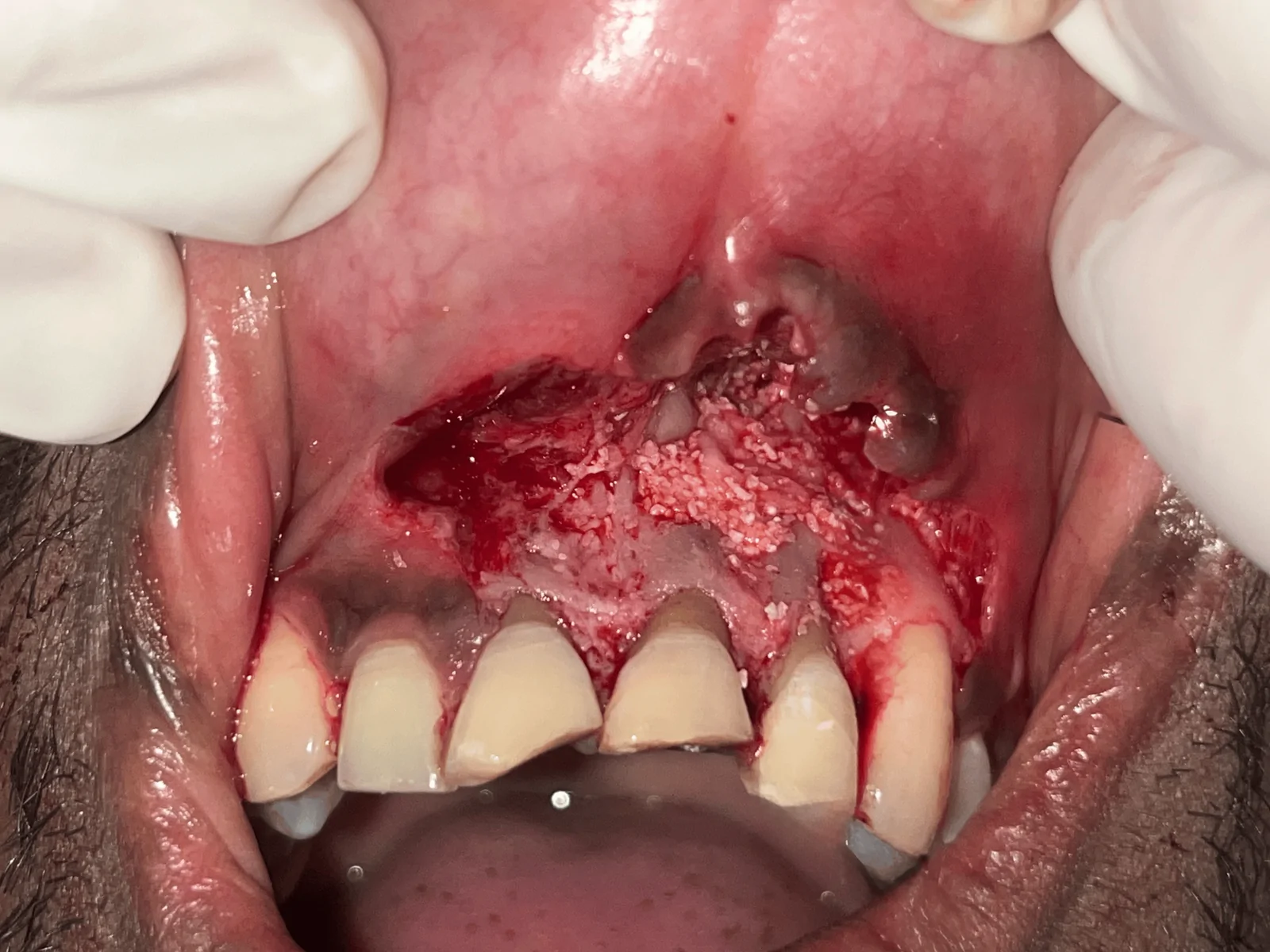
Sticky bone was prepared by combining injectable platelet-rich fibrin (I-PRF) with osseograft bone graft material for regenerative reconstruction of the osseous defect.

Leukocyte platelet-rich fibrin (L-PRF) membranes were prepared at 3000 rpm for 10 minutes. The osseous defect was filled with the graft-platelet-rich fibrin (PRF) complex and covered with a Healiguide® (Advanced Biotech Products Pvt. Ltd., Chennai, India) barrier membrane (Figure 11).

**Figure 11:**
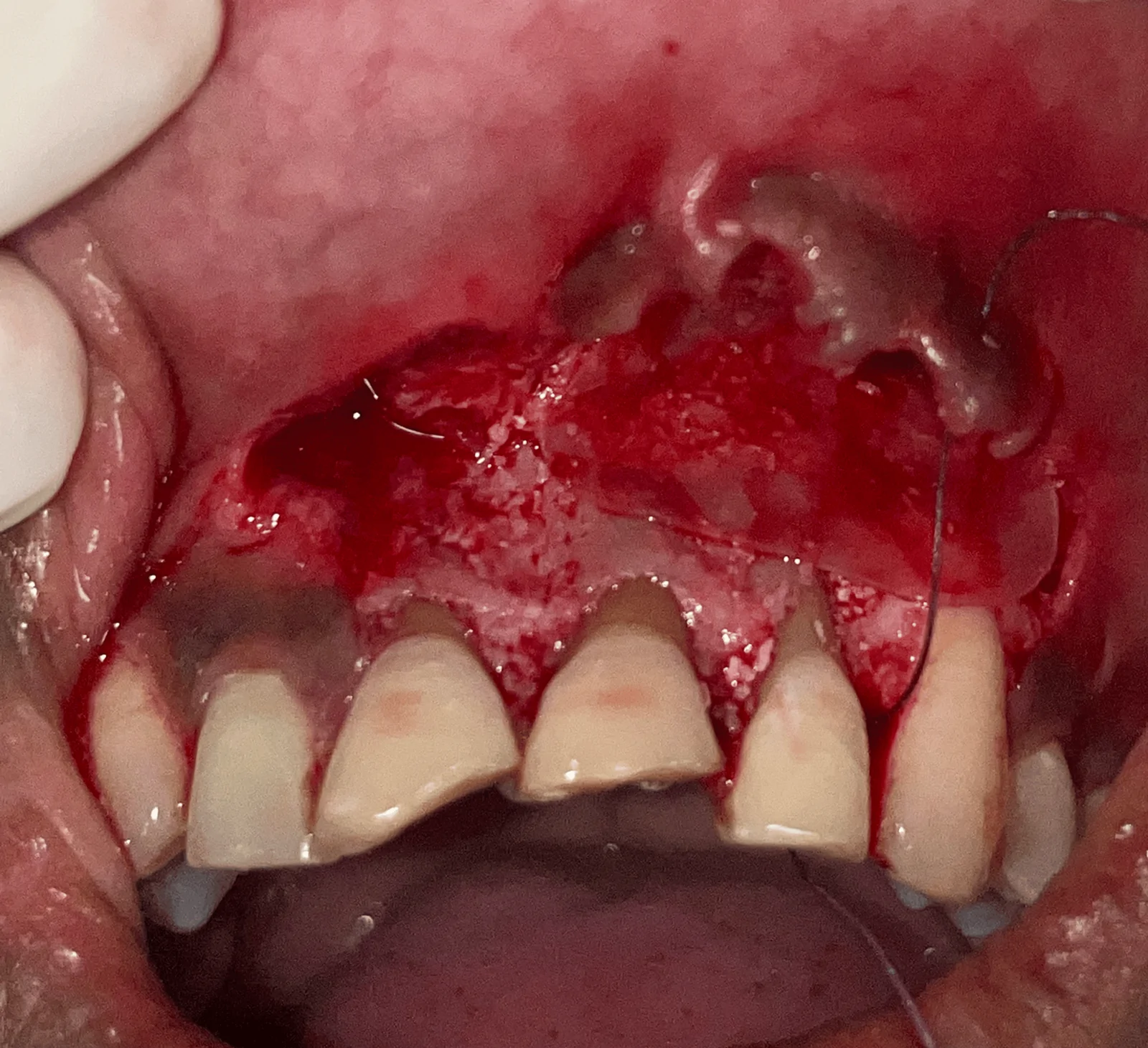
Intraoperative view showing placement of the graft–platelet-rich fibrin complex within the periapical osseous defect and coverage with a barrier membrane for guided tissue regeneration.

The flap was repositioned and secured using 4-0 Vicryl (Ethicon Inc., Somerville, NJ) sutures (Figure 12).

**Figure 12:**
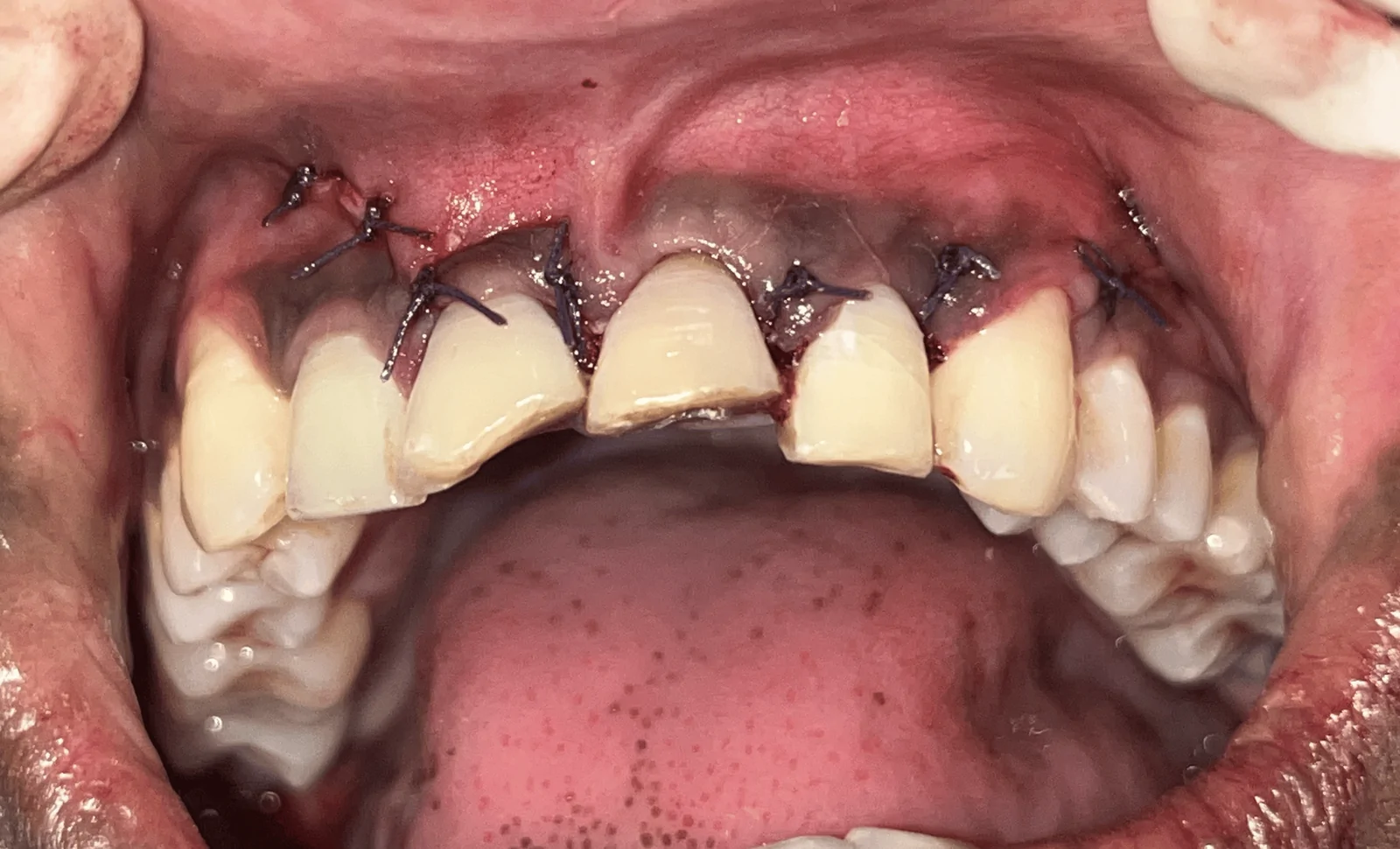
Intraoperative clinical photograph showing flap repositioning and closure of the surgical site using 4-0 Vicryl sutures.

Postoperative medications included Sporidex CV (cefalexin 375 mg and clavulanic acid 125 mg) at 12-hour intervals for five days, Metrogyl ER (metronidazole 600 mg) every 12 hours for five days, Lysoflam (diclofenac 50 mg, paracetamol 325 mg, and trypsin-chymotrypsin 50,000 AU) twice daily for five days, and pantoprazole 40 mg twice daily for five days. The patient was additionally advised to rinse with 0.2% chlorhexidine gluconate mouthwash twice daily for 15 days. Postoperative instructions regarding oral hygiene maintenance and dietary precautions were provided, and healing was monitored during subsequent follow-up visits. Sutures were removed three weeks postoperatively, following satisfactory soft-tissue healing.

Following satisfactory clinical and radiographic healing, esthetic and functional rehabilitation of the maxillary anterior teeth was undertaken. Composite post-endodontic restorations were initially placed, followed by tooth preparation for definitive full-coverage restorations. Digital intraoral scanning, bite registration, and virtual impression procedures were performed, and the digital data were transferred to the laboratory for fabrication of the definitive prosthesis. A 3D-printed cast was used to evaluate the path of insertion, occlusion, and prosthesis design. Digitally fabricated provisional restorations were tried in, adjusted, and cemented using non-eugenol temporary cement (Freegenol, GC Corporation, Tokyo, Japan) (Figure 13).

**Figure 13:**
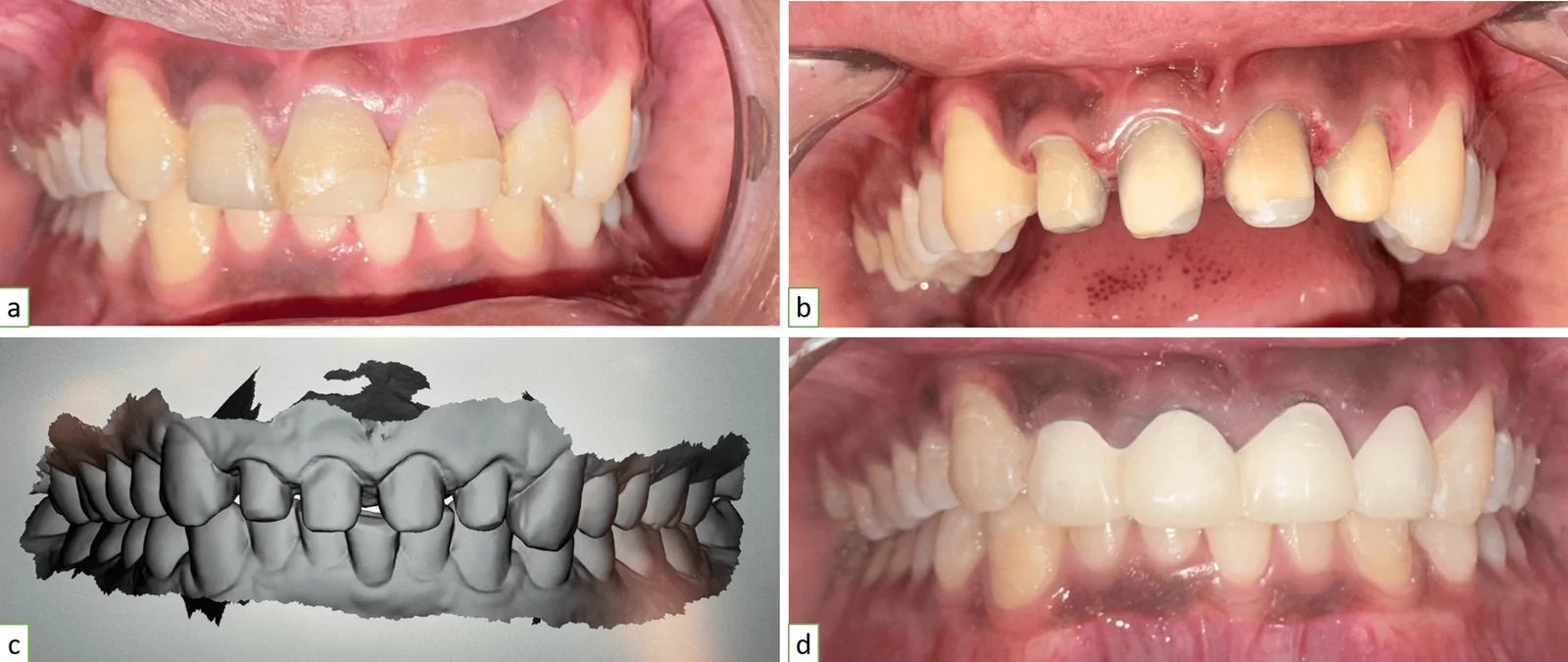
Prosthetic rehabilitation workflow. (a) Composite post-endodontic restorations following completion of endodontic treatment. (b) Tooth preparation for definitive full-coverage restorations. (c) Digital workflow comprising intraoral scanning, virtual impression procedures, and evaluation of the prosthesis on a 3D-printed cast. (d) Try-in and cementation of digitally fabricated provisional restorations.

During the definitive prosthesis appointment, the lithium disilicate (IPS e.max) full-coverage crowns were evaluated for marginal fit, internal adaptation, interproximal contacts, occlusion, esthetics, and shade matching. Intaglio surfaces were etched with 5% hydrofluoric acid for 20 seconds, followed by rinsing, drying, and silanization, and the crowns were cemented using RelyX U200 self-adhesive resin cement (3M ESPE, St. Paul, MN) (Figure 14). Residual luting cement was removed, and final finishing procedures were completed.

**Figure 14:**
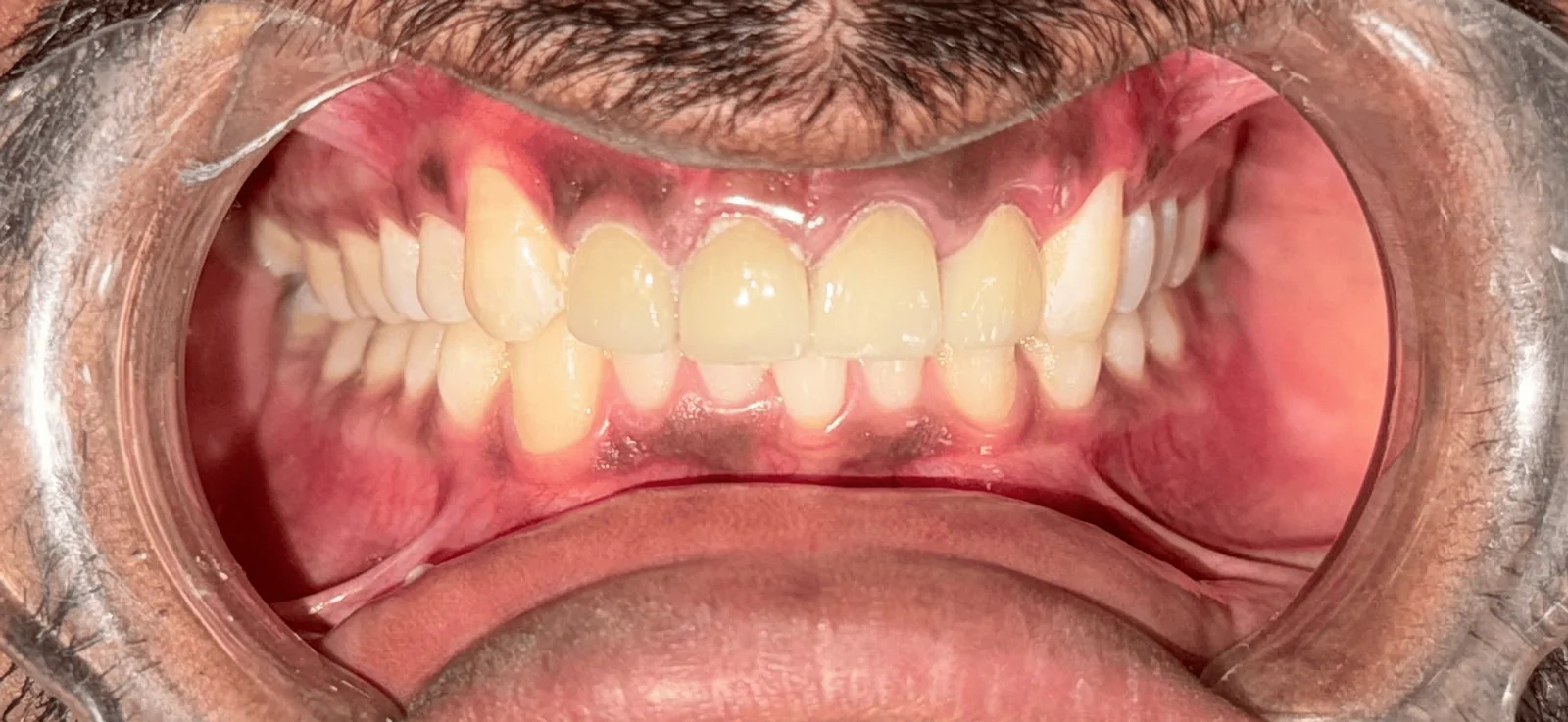
Definitive cementation of the lithium disilicate (IPS e.max) full-coverage restorations using RelyX U200 self-adhesive resin cement.

Clinical and radiographic follow-up examinations demonstrated progressive healing of the periapical lesion. At the one-year follow-up, the patient remained asymptomatic, with satisfactory esthetic and functional outcomes (Figure 15) and radiographic evidence suggestive of substantial periapical bone regeneration and lesion resolution (Figure 16).

**Figure 15:**
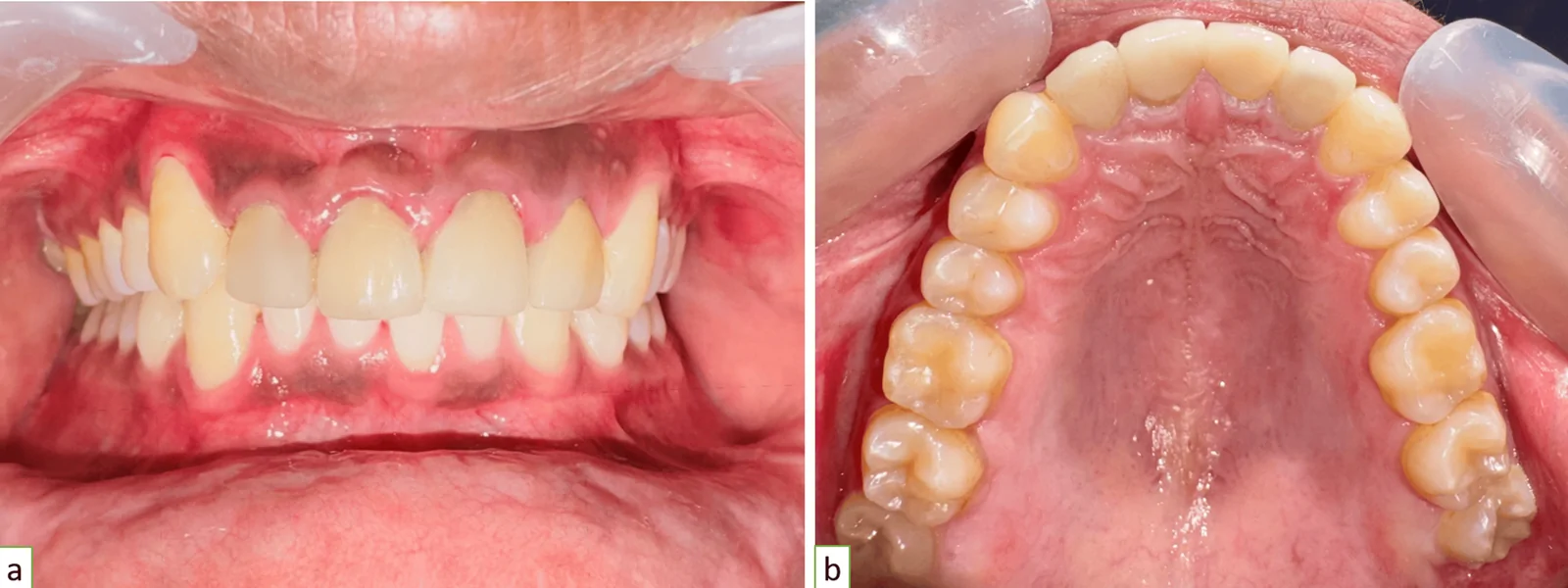
One-year postoperative clinical photograph demonstrating satisfactory esthetic and functional outcomes following definitive rehabilitation.

**Figure 16:**
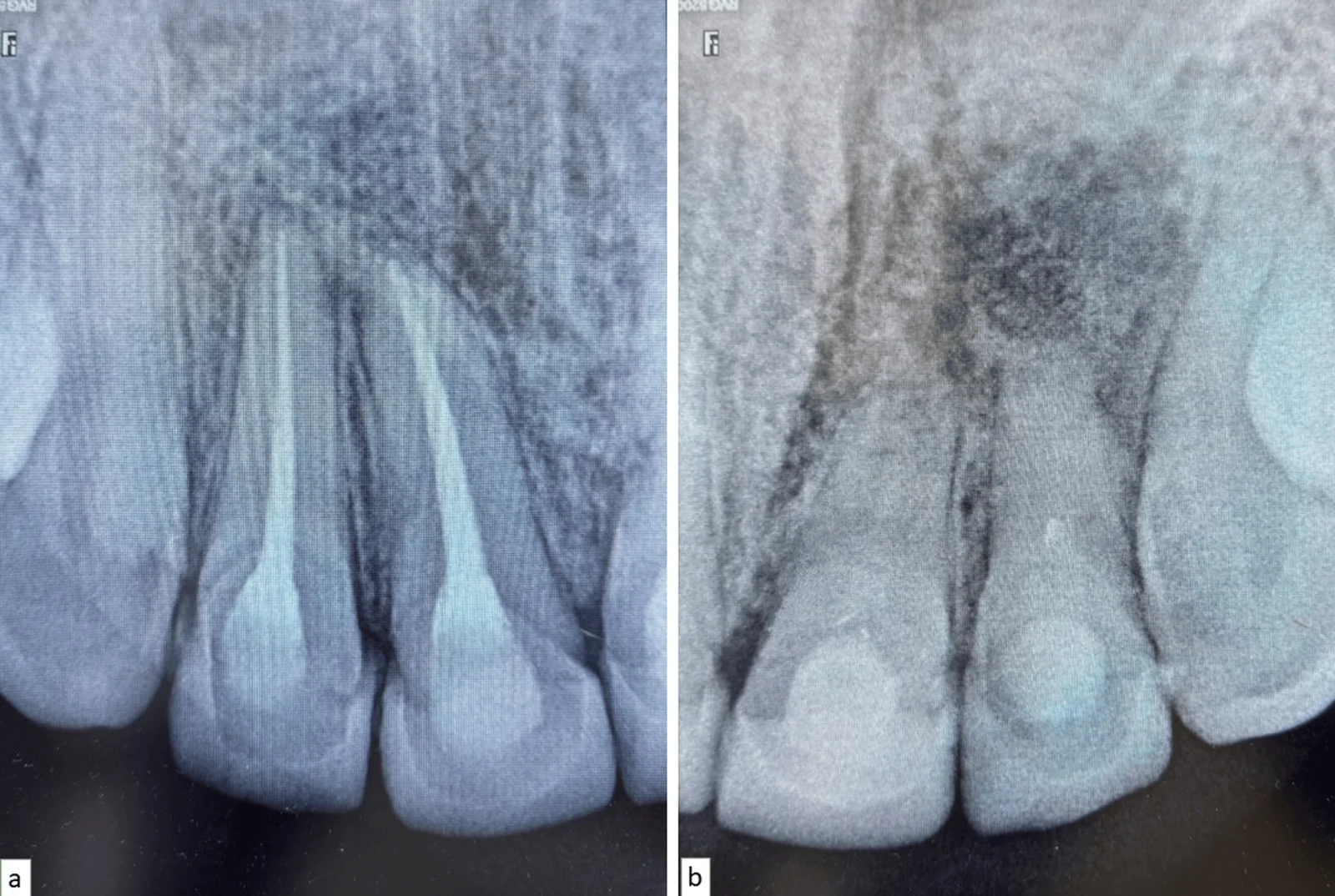
One-year follow-up radiograph demonstrating substantial periapical bone regeneration and resolution of the periapical lesion.

## Discussion

Management of large periapical lesions associated with traumatized anterior teeth remains a clinical challenge, particularly when conventional nonsurgical endodontic therapy fails to achieve resolution. Dental trauma may result in pulpal necrosis and subsequent microbial colonization of the root canal system, ultimately resulting in the development of chronic periapical pathology [8]. Although the majority of periapical lesions respond favorably to adequate chemomechanical preparation and intracanal disinfection, persistent lesions may require additional therapeutic interventions to eliminate infection and facilitate healing.

Calcium hydroxide remains a commonly employed intracanal medicament because of its antibacterial properties and ability to neutralize bacterial endotoxins. Similarly, TAP has demonstrated effectiveness against resistant endodontic microorganisms and is frequently employed in cases exhibiting persistent infection. In the present case, repeated calcium hydroxide and TAP dressings failed to achieve complete resolution of the lesion, and persistent exudation was observed during subsequent visits. Persistent apical periodontitis may result from factors such as residual intraradicular infection, extraradicular infection, foreign-body reaction, or true cystic lesions, which may compromise healing after conventional root canal treatment [9]. Extraradicular biofilms and persistent periapical infection may further limit the effectiveness of intracanal disinfection alone, thereby justifying surgical intervention in selected cases [10]. The persistent nature of the lesion in the present case ultimately necessitated surgical management.

Surgical intervention is indicated when persistent periapical pathology fails to respond to appropriate nonsurgical treatment and when direct access to the lesion is required to facilitate healing. Contemporary endodontic surgery enables thorough debridement of pathological tissues, elimination of potential extraradicular irritants, and management of apical anatomical complexities that may not be adequately addressed through intracanal procedures alone [11]. Furthermore, recent evidence suggests that incorporating regenerative approaches into periapical surgery may enhance healing outcomes in large osseous defects and improve treatment predictability [12]. In the present case, surgical management provided direct access to the lesion, allowing complete curettage of the pathological tissue, apicoectomy, and regenerative reconstruction of the periapical defect while preserving the involved teeth.

The most distinctive aspect of the current case report was the pre-surgical use of orthograde Biodentine® obturation prior to surgical intervention. Biodentine® is a calcium silicate-based bioactive material characterized by superior sealing ability, biocompatibility, bioactivity, and the ability to facilitate reparative hard tissue formation [13]. Although Biodentine® has been extensively utilized as a retrograde root-end repair material during endodontic surgery, reports describing its definitive orthograde placement before surgical intervention remain relatively limited in the literature [14]. In the present case, orthograde obturation with Biodentine® was deliberately selected to reduce surgical complexity, minimize operative time, improve patient comfort, and eliminate the risk of blood-field contamination associated with retrograde cavity preparation and filling. The favorable clinical and radiographic outcome observed at the one-year follow-up suggests that orthograde Biodentine® obturation may represent a predictable and biologically acceptable treatment option in carefully selected cases requiring surgical endodontic intervention.

Regenerative management of large periapical defects has gained considerable attention in recent years because of its potential to enhance both hard- and soft-tissue healing. PRF is an autologous biomaterial enriched with growth factors, cytokines, and leukocytes that promote angiogenesis, cellular proliferation, and tissue regeneration [15,16]. In the present case, incorporation of Osseograft® bone graft, I-PRF, L-PRF, and a Healiguide® barrier membrane helped to optimize the regenerative environment within the osseous defect. The incorporation of I-PRF with particulate bone graft facilitated the formation of a sticky bone, which enhanced graft stability and handling characteristics, while L-PRF membranes served as a biologically active scaffold that supported wound healing and tissue maturation [16]. Moreover, barrier membrane-based guided tissue regeneration has been reported to facilitate healing of extensive periapical defects by restricting soft-tissue migration into the defect and maintaining a biologically favorable space for bone regeneration [17]. The favorable radiographic evidence of bone fill and lesion resolution observed during follow-up may be attributed, at least in part, to the synergistic regenerative effects of these adjunctive therapies.

An additional strength of the present case was the comprehensive rehabilitation approach adopted following successful management of the periapical pathology. Once satisfactory healing of the surgical site was achieved, definitive prosthetic rehabilitation was undertaken to restore esthetics, function, and structural integrity of the traumatized maxillary anterior teeth. Successful management of traumatic dental injuries extends beyond elimination of infection and resolution of pathology and should also aim to restore the patient's functional and esthetic needs. In the present case, digital prosthetic workflow, including intraoral scanning, computer-aided design/computer-aided manufacturing (CAD-CAM)-assisted fabrication, and definitive adhesive cementation, facilitated predictable rehabilitation of the affected teeth. Favorable clinical and radiographic findings observed at the one-year follow-up demonstrated successful lesion resolution, substantial periapical bone regeneration, absence of clinical symptoms, and satisfactory esthetic and functional outcomes [18]. A limitation of the present report is its inherent nature as a single-case study, which restricts the generalizability of the findings. In addition, although favorable clinical and radiographic outcomes were observed at the one-year follow-up, longer-term follow-up and larger clinical studies are required to further validate the predictability and long-term success of orthograde Biodentine® obturation combined with regenerative surgical management for extensive radicular cysts.

## Conclusions

Persistent radicular cysts associated with traumatized teeth may remain refractory to conventional nonsurgical endodontic therapy despite repeated intracanal medication. In such situations, surgical intervention may provide a predictable tooth-preserving treatment option. The present case demonstrated successful intervention of a persistent periapical lesion through the use of orthograde Biodentine® obturation combined with regenerative surgical therapy utilizing platelet concentrates, bone grafting, and guided tissue regeneration principles. Favorable clinical and radiographic healing observed at the one-year follow-up suggests that this approach may be a viable alternative in selected cases where nonsurgical treatment fails to achieve resolution. Furthermore, definitive prosthetic rehabilitation contributed to the restoration of esthetics and function, resulting in a satisfactory overall treatment outcome.
